# Diffuse Idiopathic Pulmonary Neuroendocrine Cell Hyperplasia (DIPNECH): An Underrecognized Cause of Multiple Lung Nodules and Mosaic Perfusion Pattern

**DOI:** 10.5334/jbsr.2918

**Published:** 2022-11-09

**Authors:** Stijn Marcelis, Annemiek Snoeckx, Naïm Jerjir

**Affiliations:** 1 UZA, BE; 2 AZ Vitaz, BE

**Keywords:** DIPNECH, chest, nodule, CT, metastasis

## Abstract

**Teaching Point:** Diffuse idiopathic pulmonary neuroendocrine cell hyperplasia (DIPNECH) is an underrecognized and misdiagnosed cause of multiple lung nodules in combination with mosaic attenuation.

## Case History

**Figure 1:**
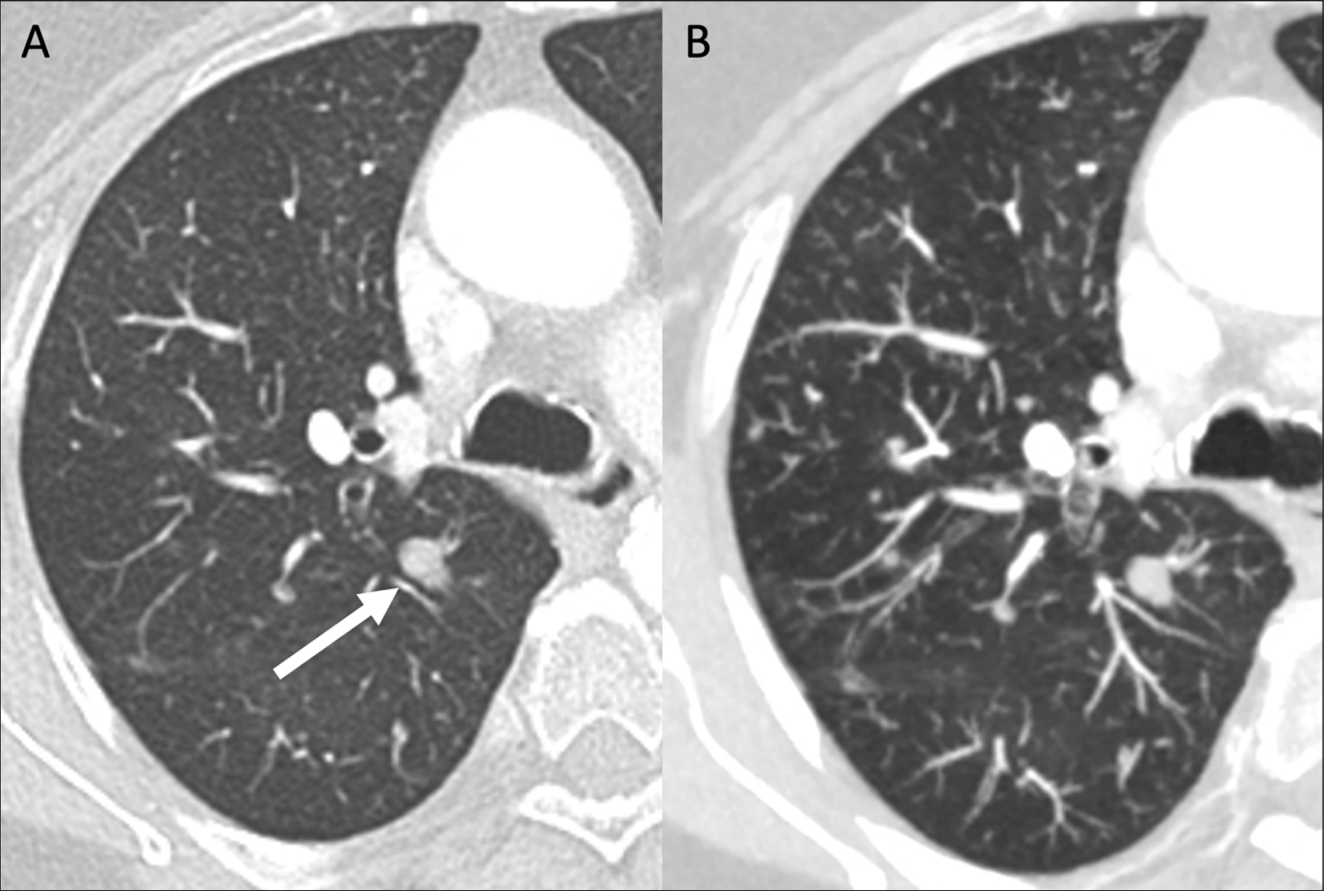


**Figure 2:**
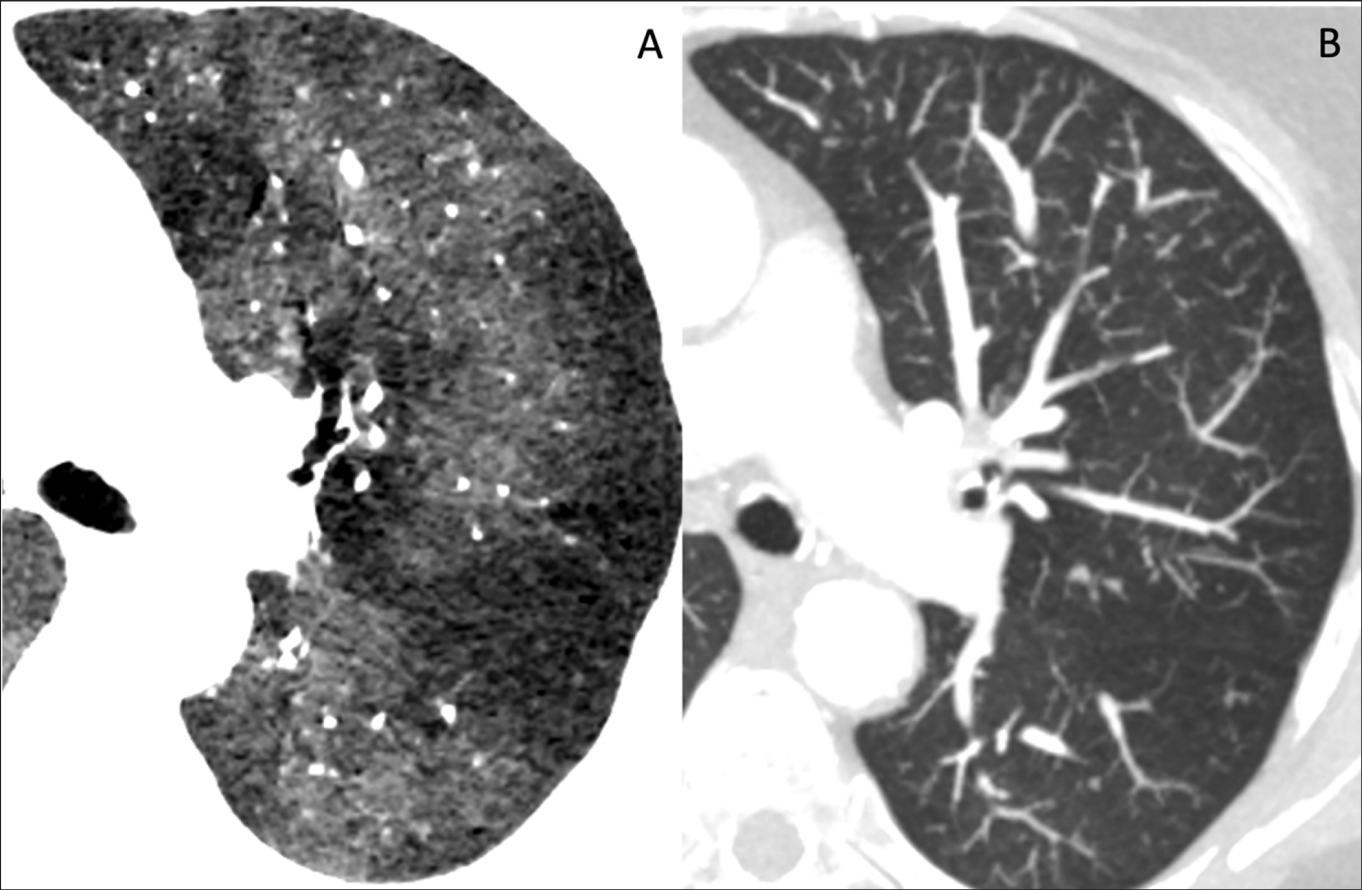


A 73-year-old woman consulted the pulmonologist for chronic cough. Lung function tests showed a moderate obstructive pattern. Computed tomography (CT) of the chest revealed multiple solid parenchymal nodules in close contact with the proximal bronchi (Figure 1a, white arrow). Tens of inconspicuous millimetric nodules became evident on maximal intensity projection (MIP) images (Figure 1b). Minimal intensity projection (MinIP) images showed mosaic perfusion pattern with relative paucity and reduced size of the (sub)segmental pulmonary arteries in the areas of reduced attenuation (Figure 2a–b). Histopathologic examination showed diffuse neuro-endocrine hyperplasia with multiple tumorlets and carcinoid tumor, diagnostic of diffuse idiopathic pulmonary neuroendocrine cell hyperplasia (DIPNECH).

## Comment

Diffuse idiopathic neuroendocrine cell hyperplasia (DIPNECH) is caused by proliferation of pulmonary neuro-endocrine cells (PNECs) or Kulchitsky cells in the epithelium of the bronchial wall, not extending beyond the basement membrane. When proliferation of PNECs extend beyond the basement membrane, they are called tumorlets or carcinoid tumors depending on the size (tumorlet < 5 mm; carcinoid tumor ≥ 5 mm).

DIPNECH is typically seen in middle-aged woman presenting with chronic cough and/or dyspnea. The diagnosis is often delayed due to clinical misdiagnosis as symptoms can mimic asthma. Radiologists should be aware of this disease as they can be the first to suggest it.

CT imaging shows bilateral, small, nodules which are more readily visible on MIP images and correspond to tumorlets or carcinoid tumors depending on their size like mentioned above. The nodules have a smooth border and may appear with a random distribution at first glance; however, distribution in DIPNECH is peribronchial since the lesions arise from the Kulchitsky cells. In contrary to multiple hematogenous lung metastases, which have a true random distribution and are the most important differential diagnosis. As practical tip, peribronchial distributed nodules in DIPNECH will not affect the fissures in contrary to hematogenous metastases.

Mosaic perfusion is a predominant but non-specific sign in DIPNECH, reported in 100% of cases. This is caused by constrictive bronchiolitis (due to secretion of peptides by PNEC hyperplasia) or proliferation of PNECs with small airway obstruction and reflectory vasoconstriction in regions of insufficient oxygenation. MinIP images are contributive to diagnose mosaic perfusion as they highlight different attenuation in the lung parenchyma. Often a nodule can be found the center of region with hypo-attenuation as they cause airway obstruction.

The final diagnosis is made on histological examination. Several treatment options are reported but the main purpose is symptomatic treatment.

DIPNECH is a preneoplastic condition where small tumorlets can evolve into typical or atypical carcinoid tumors. In rare cases lymph node metastasis or adrenal gland metastasis can develop. Therefore, long-term follow-up with CT is recommended [1].
